# Modified Borggreve–Van Nes-Winkelmann rotationplasty for surgery in developing countries

**DOI:** 10.1186/s12893-022-01780-z

**Published:** 2022-09-07

**Authors:** Laura Sommerauer, Aung Phyo, Eric Pion, Isabel Zucal, Eric Klingelhoefer, Si Thu, Than Win, Sopyay Khin, Thura Kyaw, Hein Htet Zaw, Maung Mg Htwe, Nicola Fabbri, Silke Haerteis, Thiha Aung

**Affiliations:** 1 Centre of Plastic, Aesthetic, Hand and Reconstructive Surgery, Clinic Traunstein, Traunstein, Germany; 2 Sarcoma and Musculoskeletal Oncoplastic Division, Department of Orthopaedic Surgery, University of Medicine, Mandalay, Myanmar; 3 Institute for Molecular and Cellular Anatomy, University of Regensburg, Regensburg, Germany; 4 Surgery Department, Kantonsspital Aarau, Tellstrasse 25, 5001 Aarau, Switzerland; 5 Department of Plastic, Aesthetic and Reconstructive Microsurgery, Specialized Burn Center, Trauma Center Murnau, Murnau, Germany; 6 Orthopaedic Service, Department of Surgery, Memorial Sloan Kettering Cancer Center, New York, USA; 7 Faculty of Applied Healthcare Science, Deggendorf Institute of Technology, 94469 Deggendorf, Germany; 8 Centre of Plastic, Aesthetic, Hand and Reconstructive Surgery, University Medical Centre Regensburg, Regensburg, Germany

**Keywords:** Orthopedic surgery, Plastic surgery, Reconstructive surgical procedure, Surgical oncology

## Abstract

**Background:**

Amputation is still the most common therapy for patients suffering from osteosarcoma in Myanmar, despite the fact that limb salvage surgery e.g. Borggreve–Van Nes-Winkelmann rotationplasty for malignant tumors located within the distal femur or proximal tibia is the current state-of-the-art reconstructive procedure. A safe and reliable operation technique is crucial in order to perform a complex surgical procedure like the rotationplasty in lower-middle income economies with limited infrastructure and resources. The authors present seven cases of patients with osteosarcomas that received a Borggreve–Van Nes-Winkelmann rotationplasty with an evaluation of the procedures focusing on safety and sustainability.

**Methods:**

From 2019 until 2020, seven young patients with osteosarcomas of the distal femur or proximal tibia were treated with Borggreve–Van Nes-Winkelmann rotationplasties in the Orthopaedic Hospital in Mandalay, Myanmar. As modification of the standard procedure the dissection and subsequent clamping of the femoral artery in order to minimize blood loss as well as the formation of an adipocutaneous flap that minimizes swelling and decreases the pressure on the vessels were successfully performed. This modified procedure resembles a safe and simplified surgical technique that is feasible under the circumstances of lower-middle income economies with good outcomes.

**Results:**

All patients showed good functional and aesthetic results. One of the seven patients needed secondary wound closure due to wound dehiscence.

**Conclusions:**

A simplified and safe operation technique for the performance of the Van Nes-Borggreve rotationplasty was adapted to the given constraints in lower-middle income economies and proved to be successful.

*Trial registration* All patients approved to participate in the study and have given consent to publication.

## Introduction

The treatment of patients suffering from limb malignancies is challenging, especially in lower-middle income economies with suboptimal clinical circumstances, limited infrastructure and resources. Therefore, amputation is still the most common therapy of patients with osteosarcomas located in the lower extremities in lower-middle income economies even though limb-salvage procedures have become the state-of-the-art surgical therapy due to the rapid progress in musculoskeletal oncology [1]. Regarding these limb-salvage surgical procedures, Borggreve–Van Nes- Winkelmann rotationplasty is a popular option which is currently performed as a standard therapy in high-income economies as part of a multidisciplinary approach with good outcomes for overall disease survival [2, 3].

The improvement of survival rates of osteosarcoma patients due to innovative neoadjuvant and adjuvant chemotherapy demands for an optimal recovery of function. Therefore, there is a high demand for new surgical procedures in lower-middle income economies where above-the-knee amputation is still the most common surgical treatment for distal femoral malignancies. Borggreve–Van Nes-Winkelmann rotationplasty allows an en-bloc resection of the tumor with clear margins. Hereby parts of the distal femur and proximal tibia are removed while keeping the neurovascular structures intact. The remaining part of the lower leg including the foot is rotated around 180° in the axial plane and attached to the remaining proximal part of the femur stump. Requirements for this procedure are a disease-free foot and ankle, intact nerves and a maintainable or restorable vascular supply of the ankle [4, 5].

This technique was first described by Borggreve in 1930 for a patient with severe shortening of the leg after tuberculosis of the knee [6].Subsequently, this procedure was also performed as a surgical management of congenital femoral dysplasia by Demel and Gold (1932) [7] and Van Nes (1950) [8].The first research work regarding rotationplasty as a possible operative procedure for the treatment of bone malignancies of the knee region was published by Salzer in 1976 [9]. In the early ’80s, Winkelmann [10, 11] modified the surgical technique, so that not only tumors of the distal femur, but of the entire femur, the lower pelvic region and the proximal to mid tibia could be operated via rotationplasty. Winkelmann also established a classification for rotationplasty surgery by focusing on the localization of the tumor in the distal femur (type AI) or the proximal tibia (type AII) [12]. In comparison to the treatment of osteosarcomas of the distal femur via proximal femoral amputation, which requires an upper leg prosthesis, rotationplasty type AI only requires a lower leg prosthesis because of the preservation of an actively mobile knee joint. Further advantages include the preservation of the highly sensitive plantar foot surface and the possibility of growth due to the sparing of the growth plates of the distal tibia in children which can compensate for length differences over time. A rotationplasty preserves the extremity even in advanced cases where other limb-salvage procedures are no longer possible such as skip metastases, inadequate soft-tissue conditions, or knee joint involvement [13].

Even though this procedure is mostly carried out in children, Gaillard et al. report an excellent outcome in a 38-year old patient who underwent more than 20 surgeries with a vast array of complications, in a time span of 25 years after the diagnosis of osteosarcoma before receiving the final rotationplasty [14]. For a complex surgical technique such as the described rotationplasty in lower-middle income economies with limited infrastructure and resources, a safe and reliable operation method is necessary. The authors present seven cases from the Orthopaedic Hospital in Mandalay, Myanmar for the evaluation of this treatment approach that focuses on a safe and sustainable treatment of osteosarcoma patients.

It has to be mentioned, that the last author of this study is originally from Myanmar and an official cooperation between the University of Regensburg and the University of Mandalay was established after numerous humanitarian missions with the organization pro Interplast Seligenstadt e.V. in Myanmar. This excellent cooperation was established for the joint training of young students and surgeons which includes joint operations in both locations for educational and research purposes.

## Patients and methods

Approval was granted by the local ethics committee of the University Medicine Mandalay, Myanmar. In February and November of 2019, as well as February 2020, seven young patients suffering from osteosarcomas of the distal femur or proximal tibia received a rotationplasty in the Orthopaedic Hospital in Mandalay, Myanmar. The patients were 9, 12 (three children), 13, 14, and 17 years of age at the time of the procedure. The surgeon responsible (i.e. the corresponding senior author of this paper) participated in the very first case of a rotationplasty, performed on a 7-year-old patient with chondromyxoid fibroma of the left distal femur in November 2018 in Myanmar. We now present the following seven patients that received a Borggreve–Van Nes-Winkelmann rotationplasty for the treatment of limb malignancies. The operations were performed by the corresponding and senior author of this article and the local orthopedic team in the Mandalay Orthopaedic Hospital. In contrast to the standard procedure, the dissection and subsequent clamping of the femoral artery which minimizes blood loss as well as the dissection of an adipocutaneous flap that minimizes swelling and decreases the pressure on the vessels were performed. This additional flap enables a better coverage of the larger blood vessels and prevents the formation of seromas in the cavities. These modifications resemble a safe and simplified surgical technique tailored to the setting of a lower-middle income economy. The outcomes and complications were evaluated with regards to safety and sustainability.

The minimal follow-up of these patients was based on the standard pediatric oncological follow up according to the German S1 guidelines. These include postoperative chest X-ray in addition to X-rays of the primary tumor location and diagnostics for long-term effects of the disease. The prognosis for patients suffering from osteosarcoma with relapsed disease after surgery is very poor which is why amputation appears to be the last treatment possibility in this course of disease. Specific action regarding other functional postoperative complications were not previously determined but would have depended on the individual situation. However, no disease relapse occurred.

## Results

One female and six male patients diagnosed with osteosarcoma of the lower limb received a rotationplasty at the Orthopaedic Hospital in Mandalay, Myanmar in February and November of 2019 as well as February 2020. Early postoperative outcomes showed good functional and aesthetic results. One patient experienced wound dehiscence due to excessive tension that occurred as a postoperative minor complication. The patient was brought to the operation room for secondary wound closure which was followed by an uneventful postoperative healing process. Follow-up care was provided by the orthopedic and oncological team of Mandalay Orthopaedic hospital in Myanmar and by the corresponding author via regular phone and video calls with the patients and their families.

### Case series

All patients were admitted to Mandalay Orthopaedic Hospital for a biopsy of the distal femur or proximal tibia. The histopathological result revealed an osteosarcoma in all cases. Subsequently, the board of the Joint Cancer Clinic decided to treat the patients with a rotationplasty. The surgical procedure was performed as described and an inguinal lymph node biopsy was sent to pathology.

*Patient 1* was a 17-year-old female who was diagnosed with chondroplastic osteosarcoma of the left distal femur. She presented with pain in the left knee joint that started 4 months ago in addition to swelling in the left thigh which was noticed one month previously. The knee joint pain was located mainly at the medial side with a slow onset, dull aching in character, aggravated by movements and relieved by rest and analgesia. She had no history of night pain, fever, loss of weight or appetite. She noticed a left thigh swelling laterally in the distal 1/3 of the thigh. The swelling gradually increased over time.

*Patient 2* was a 13-year-old male who was diagnosed with osteosarcoma of the right distal femur. The patient had already received chemotherapy for three months but still showed tumor progression during chemotherapy.

**Fig. 1:**
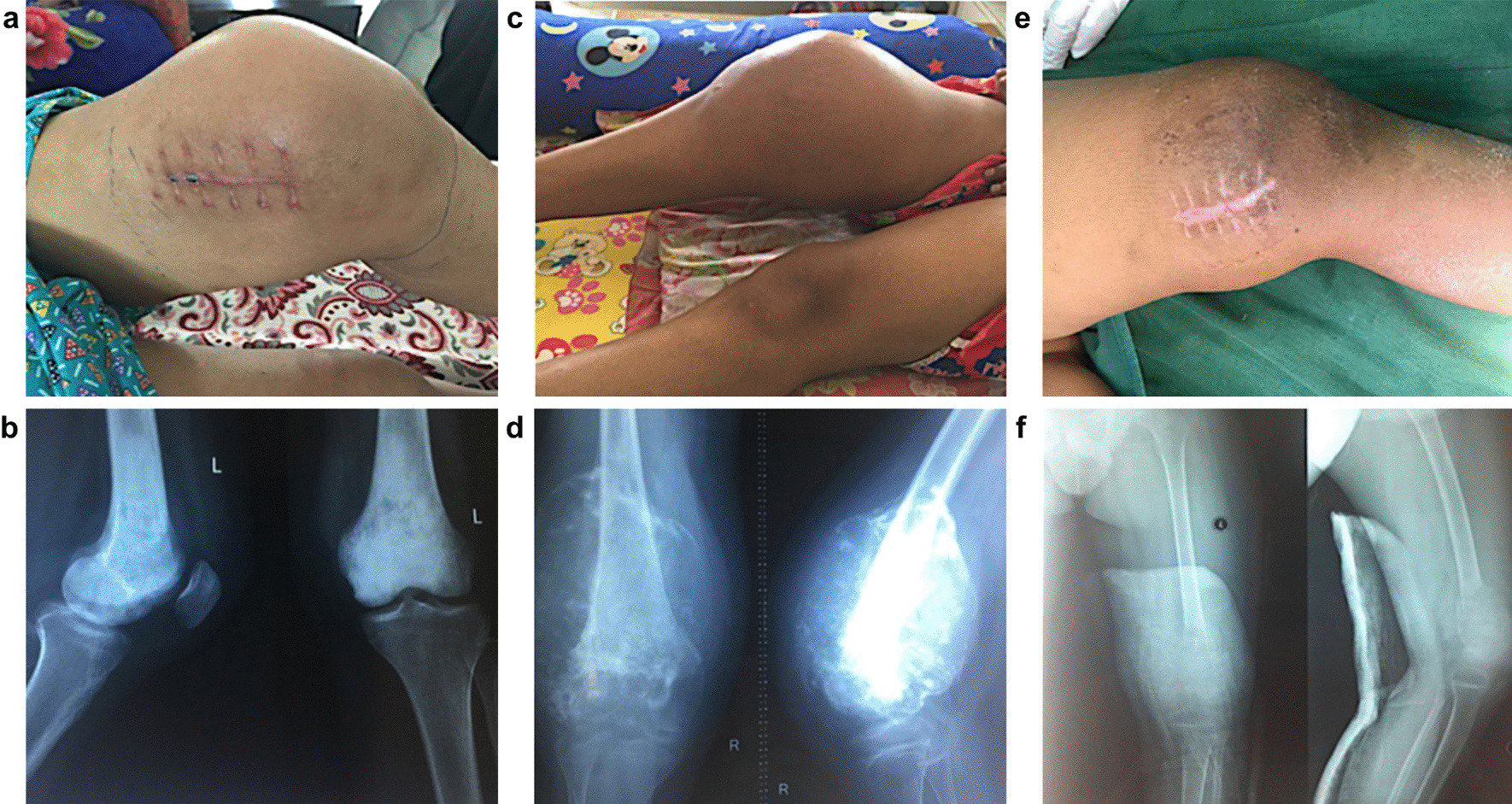
Clinical images and X-rays of patients 1–3. **a** and **b** Patient 1 with osteosarcoma of the left distal femur and corresponding X-ray; **c** and **d** Patient 2 with osteosarcoma of the right distal femur and corresponding X-ray; **e** and **f** Patient 3 with osteosarcoma of the left distal femur and corresponding X-ray

*Patient 3* was a 9-year-old male who had a pathological fracture after an accident in October 2018. A biopsy revealed an osteosarcoma of the left distal femur. Clinical images and x-rays of the patients 1–3 are provided in Fig. 1a–f.

*Patient 4* was a 12-year-old male with osteosarcoma of the left distal femur. He had received chemotherapy three times until July 2019.

*Patient 5* was a 12-year-old male with osteosarcoma of the left proximal tibia.

*Patient 6* was a 12-year-old male with osteosarcoma of the right proximal tibia.

**Fig. 2:**
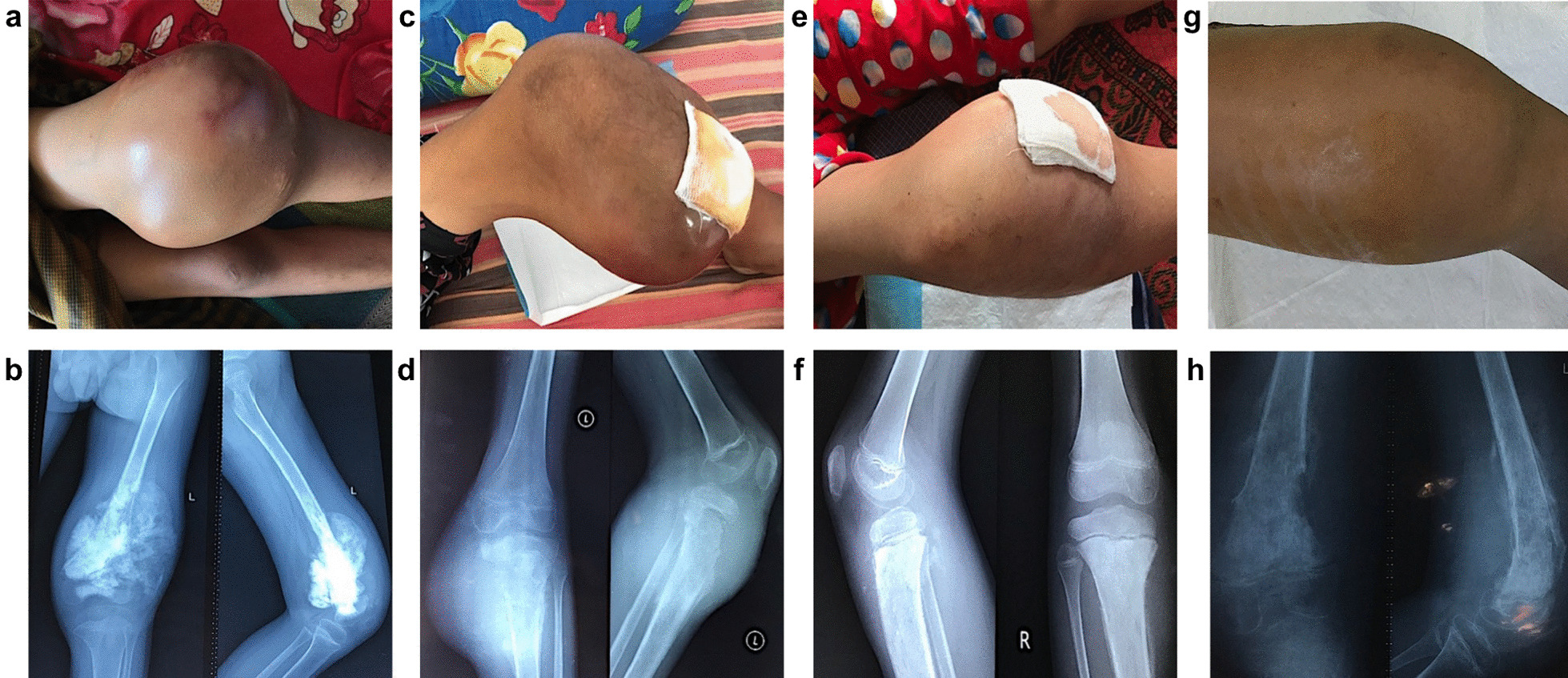
Clinical images and X-rays of patients 4–7. **a** and **b** Patient 4 with osteosarcoma of the left distal femur and corresponding X-ray; **c** and **d** Patient 5 with osteosarcoma of the left proximal tibia and corresponding X-ray; **e** and **f** Patient 6 with osteosarcoma of the right proximal tibia and corresponding X-ray; **g** and **h** Patient 7 with osteosarcoma of the distal femur and corresponding X-ray

*Patient 7* was a 14-year-old male with osteosarcoma of the left distal femur. Clinical images and X-rays of the patients 4–7 are provided in Fig. 2a–h.

### Surgical technique

#### Pre-operative preparation

Each operation was performed under general anesthesia and in a prone position with a pillow under the ipsilateral pelvis to ensure a better access for the preparation of the sciatic nerve and popliteal structures. The patients received intravenous antibiotic prophylaxis and the entire leg, including the foot, was cleaned and draped.

#### Operation technique

**Fig. 3:**
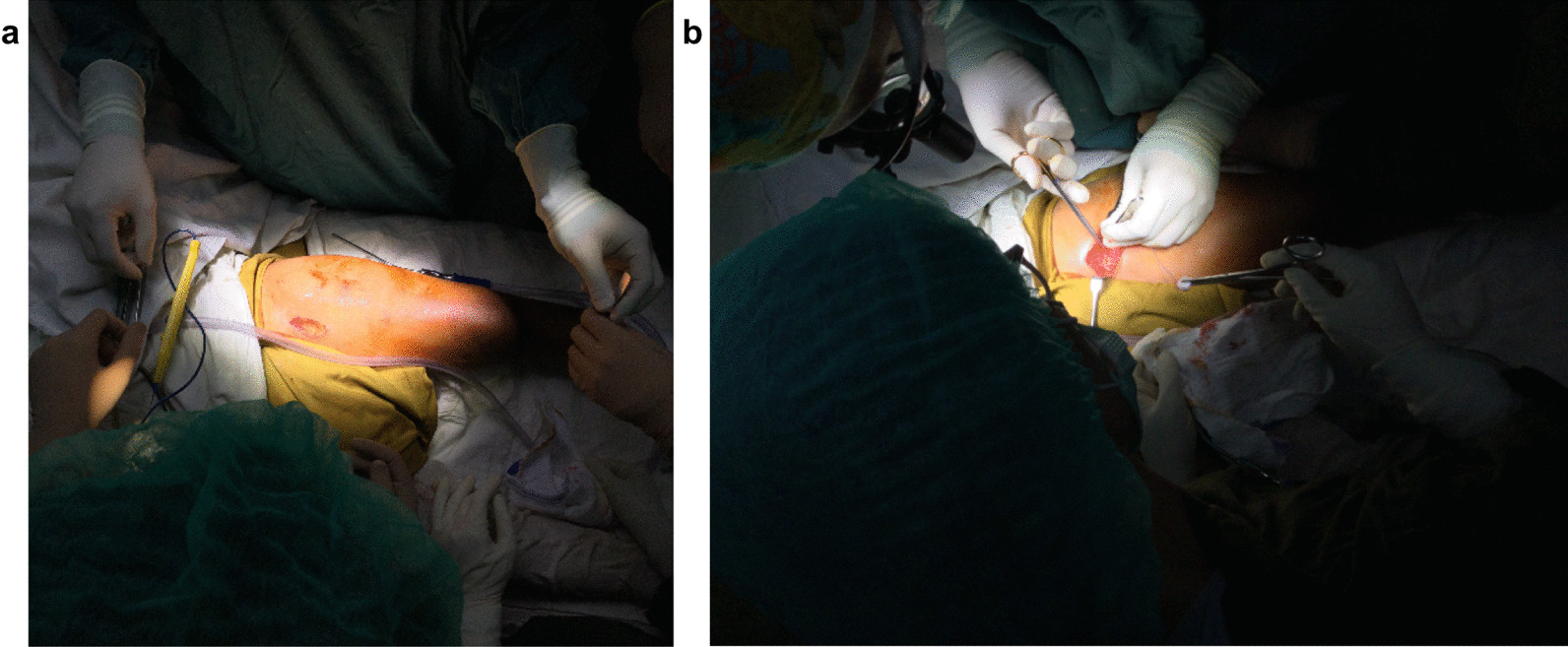
**a** First incision on the proximal medial thigh to expose the femoral artery and vein. **b** Continuous exposure of the blood vessels

First, a longitudinal incision was performed below the groin for the dissection of the femoral artery and vein (Fig. 3a and b).

**Fig. 4:**
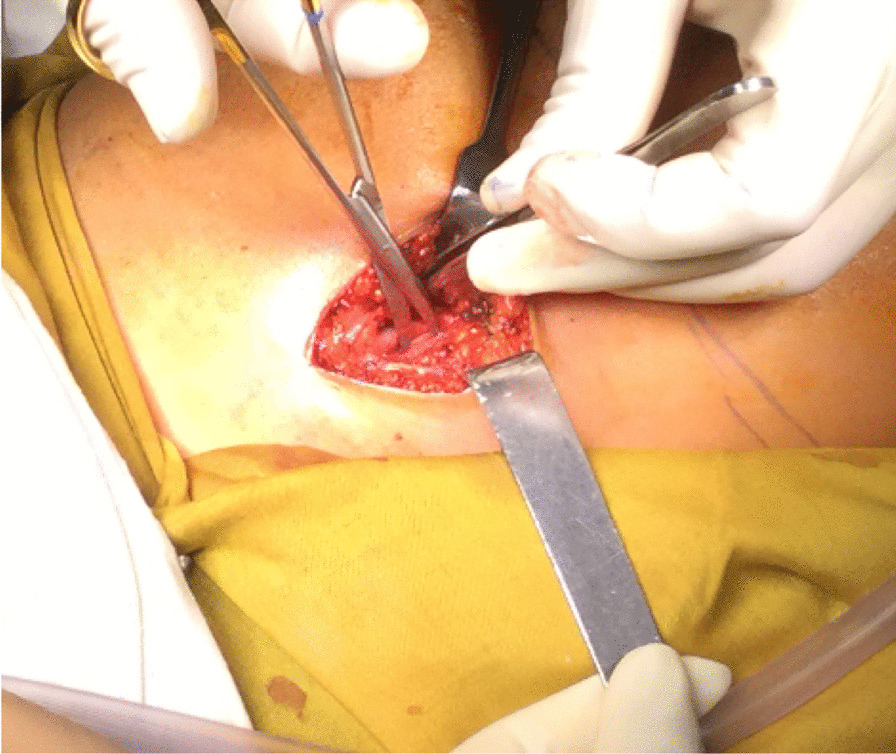
Clamping of the femoral artery and vein after dissection on proximal medial thigh region

The vessels were exposed until approximately 2 cm caudal of the lesser trochanter. Then, the femoral artery was clamped (first ischemia-time) to prevent bleeding during the operation (Fig. 4).

**Fig. 5:**
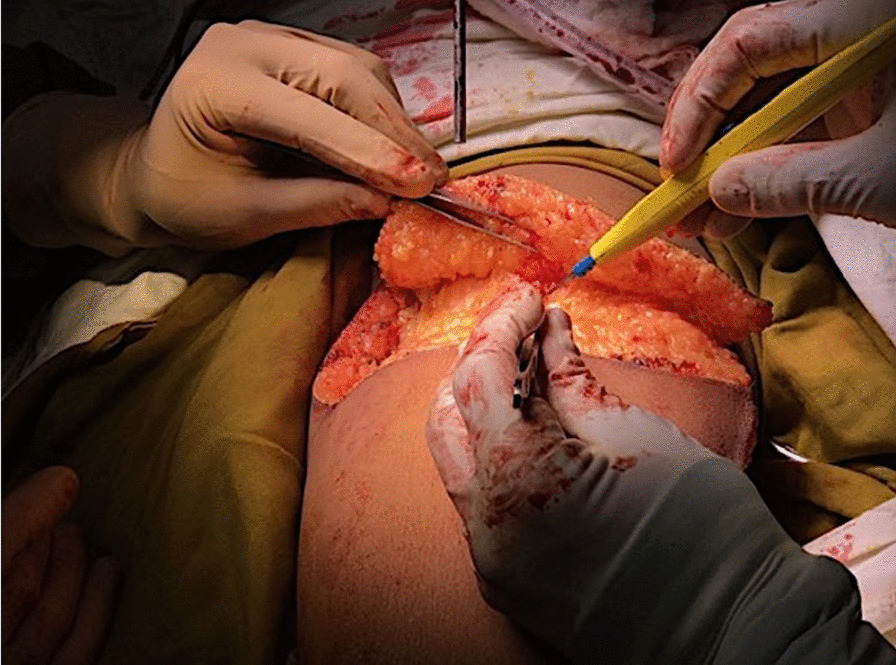
Adipo-cutaneous flap. Dissection of the circular adipo-cutaneous flap is depicted. Tissue thickness was assured to provide an optimal coverage of the osteosynthesis

The longitudinal incision from the joint was extended laterally and medially to perform an adipo-cutaneous advancement flap (Fig. 5).

**Fig. 6:**
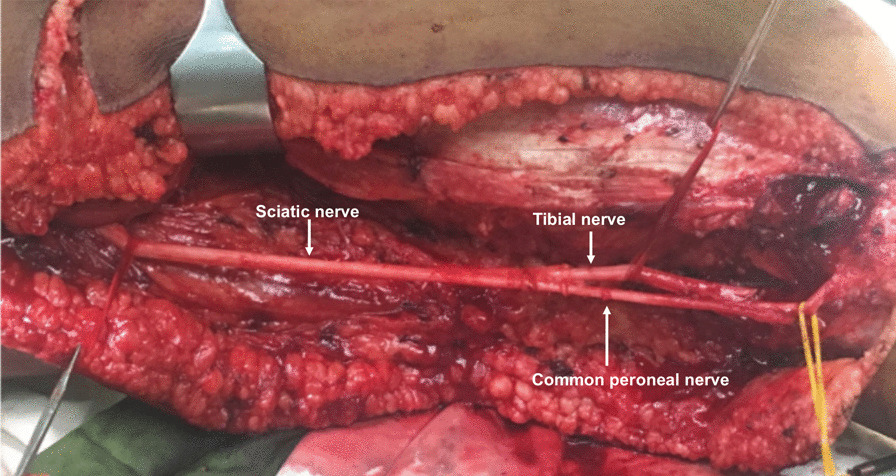
Surgical dissection of the sciatic nerve with the transition into the tibial nerve and the common peroneal nerve proximal of the popliteal fossa

The second incision was made dorsolateral and caudal to the head of the fibula (the direction of the incision followed the course of the tendon of the biceps femoris muscle) in order to expose the common peroneal nerve and lateral sural cutaneous nerve. The common peroneal nerve was then dissected proximally until its bifurcation from the sciatic nerve and continuous surgical dissection of the sciatic nerve behind the biceps femoris muscle was performed (Fig. 6).

**Fig. 7:**
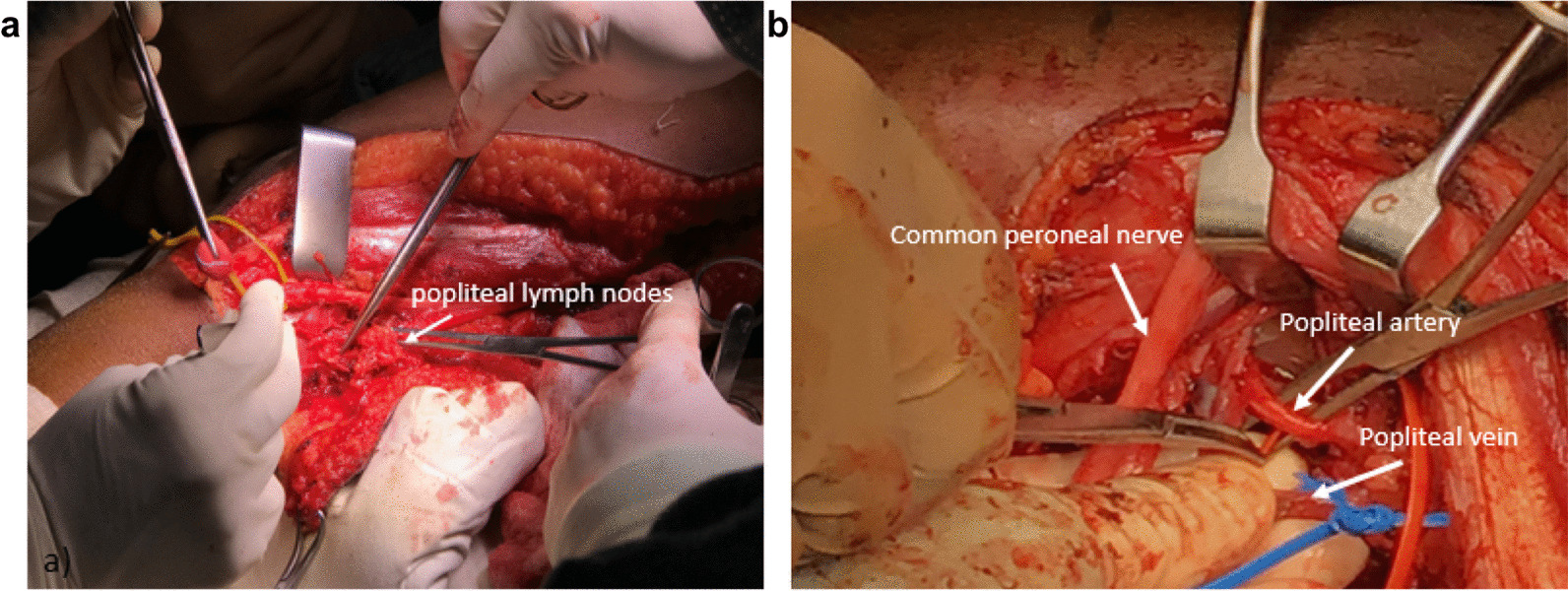
Exposure of the popliteal anatomical structures. **a** Dissection of popliteal lymph nodes for biopsy and **b** dissection of popliteal artery and vein

A third incision was made horizontally to the second incision for the exposure of the popliteal region. The popliteal artery, vein and lymph nodes were subsequently located and dissected (Fig. 7).

The fourth incision was performed circumferentially about 2 cm caudal of the tibial tuberosity. After surgical dissection of the soft tissue of the lower leg, the head of the fibula was then disarticulated from the tibiofibular joint. Furthermore, the tibia was divided caudal of the tibial tuberosity depending on the extent of the tumor while preserving the popliteal neurovascular structures. Subsequently, the proximal circumferential skin incision was made about two centimeters caudal of the lesser trochanter. A tent- or V-shaped skin envelope was dissected cranially to ensure the preservation of abundant soft tissue for the skin adaption and wound closure after the procedure. The soft tissue of the upper leg was dissected while protecting the sciatic nerve. Before the proximal osteotomy of the femur was performed, the femoral and popliteal arteries and veins were clamped (second ischemia time) and cut while keeping the stumps as long as possible.

**Fig. 8:**
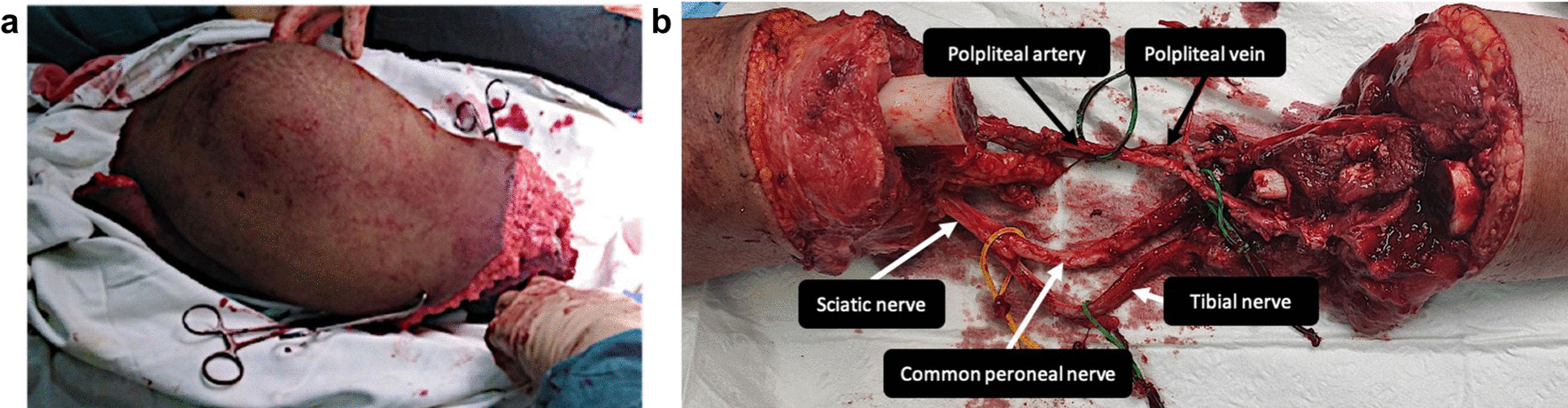
Tumor resection. **a** The resected tumor mass is shown. **b** The neurovascular structures including the popliteal vessels and the sciatic nerve were preserved

After the osteotomy of the femur and tibia was performed with sufficient margins to the healthy tissue, the en-bloc resection of the tumor was performed (Fig. 8a). Hereby, the important popliteal neurovascular structures were protected (Fig. 8b). The specimen was then sent to pathology.

**Fig. 9:**
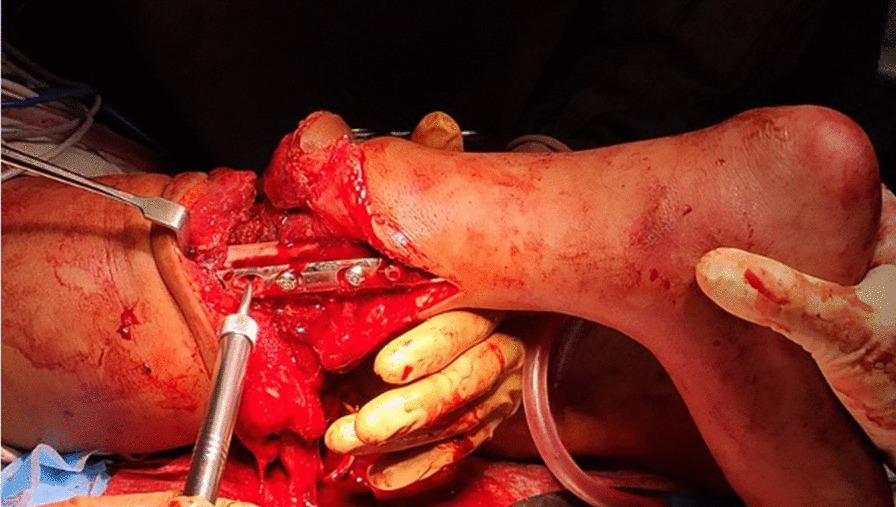
Bony reconstruction. Intraoperative image of osteosynthesis of tibia and femur with a dynamic compression plate is shown

The remaining foot and ankle were rotated 180° in the axial plane and osteosynthesis of tibia and femur was performed with a femur plate (Fig. 9).

**Fig. 10:**
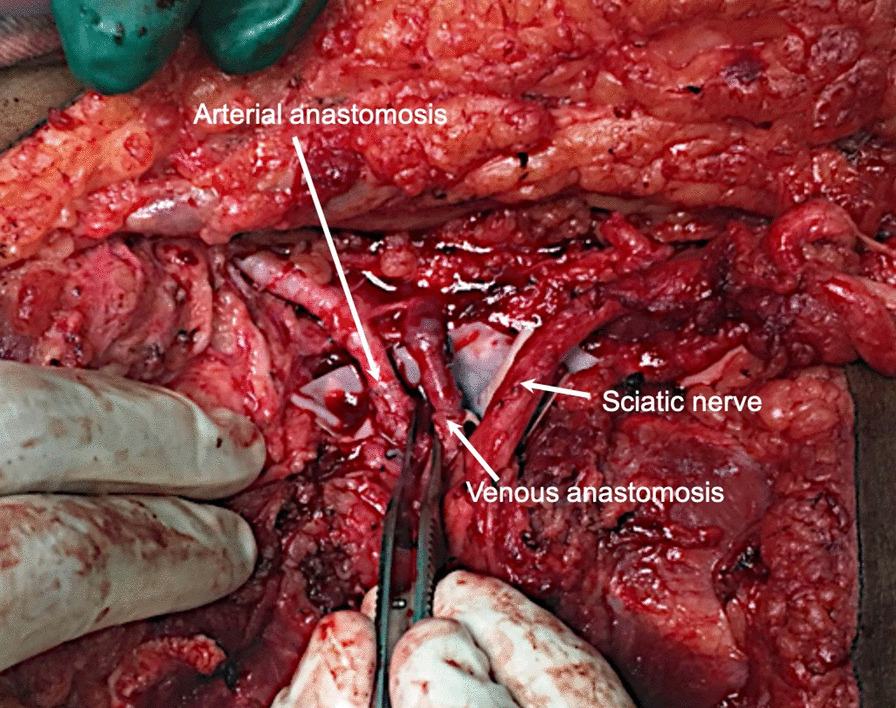
Vascular repair. End-to-end anastomoses of the femoral and the popliteal artery and vein were performed

Two vascular anastomoses (one vein and one artery) were required for sufficient blood supply of the extremity. The vessels were infiltrated with heparin solution to prevent thrombosis. The vascular anastomoses of the femoral and popliteal artery, as well as femoral and popliteal vein were performed with 8–0 sutures (Fig. 10).

**Fig. 11:**
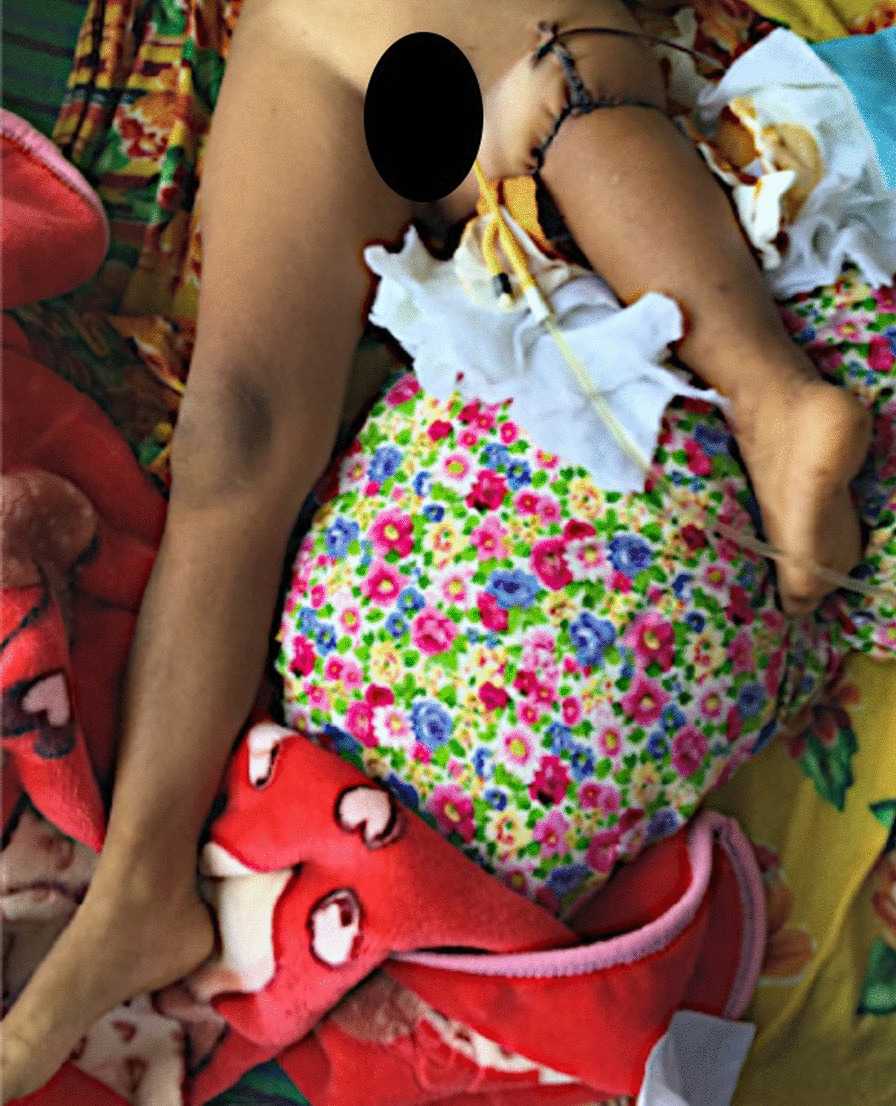
Postoperative results. A postoperative image of the reconstructed limb after osteosarcoma of the distal femur is depicted

A small pouch for the safe placement of the neurovascular structures was dissected. The sciatic nerve was safely covered underneath the circular adipo-cutaneous advancement flap in order to prevent any potential injury of the nerve. The tendon of the gastrocnemius muscle was attached to the rectus femoris muscle to enable the muscular movement of the new knee joint. Two redon drains were positioned under the fascia prior to the circular subcutaneous sutures and skin closure. The sutures were loosely placed to prevent excessive pressure on the vessels and to ensure adequate blood flow through the vascular anastomoses. Perfusion of the foot was also checked after closure. Only orthopedic wool was used as a loose bandage to prevent any pressure on the vessels. The bandages covering the foot and toes were taken off for the perfusion control (Fig. 11).

#### Post-operative care and rehabilitation

The patients were transferred postoperatively to an intensive-care unit for less than 12 h. A pulse-oximeter on one toe was used to measure the oxygen saturation in the patient’s blood. The foot was observed regularly for palpable pulse, temperature and skin color to discover any early signs of potential ischaemia (Fig. 5). Movement in the ankle joint was checked immediately after waking up from the anesthesia and was sufficiently present in all cases.

The patients as well as their family members were instructed to perform exercises and move the foot in order to prevent ankle stiffness. These consisted of different variations of flexion and extension of the ankle joint with or without resistance. This is important for the improvement of the venous circulation and for the training of the neural plasticity for the new biomechanical functions of the different muscles and joints. An orthopedic technician from Germany gave tutorials on how to prepare and adjust a prosthesis for rotationplasty patients. He attended the local orthopedic workshop and performed a hands-on-training for local orthopedic technicians for the precise modeling of these special prostheses.

## Discussion

Surgical interventions in lower-middle income or even low-income economies with the aim of conducting successful orthopedic surgeries (e.g. cleft palate surgery, pediatric surgery) have been previously described in the literature and carry certain difficulties regarding organization, costs, time and personnel next to many other variables [15, 16]. The success of such incentives is based on adequate organization in advance together with the local team of surgeons, experience, adaptability and Non-profit organizations and their donors. With regards to osteosarcoma as a specific disease, good results were reported by Pan et al. for limb-salvage surgery using tumor prostheses in an upper-middle income economy [17]. However, this study was conducted by a local team of surgeons which enabled an easier acquisition of patients next to a more extensive follow-up.

Limb-salvage surgery is currently the gold standard surgical procedure for the treatment of osteosarcomas of the lower limb region. Possible treatment options include Borggreve–Van Nes-Winkelmann rotationplasty, allografts, autografts, the use of devitalized tumor-bearing bone and the Masquelet technique [18–21]. Borggreve–Van Nes-Winkelmann rotationplasty is mostly used for children with open epiphyseal plates that enable them to grow but simultaneously inhibit the performance of other types of limb salvage surgery options since these may not work well. Regarding this rotationplasty, the femur and the tibia will continue to grow as the child gets older and the prosthesis can be extended in proportion to the patient’s growth. Furthermore, it is easier for younger children to adapt their control of the motor functions to use the former ankle joint as a novel knee joint and to adapt their walking patterns accordingly.

Advantages of Borggreve–Van Nes-Winkelmann rotationplasty also include the possibility of a wide tumor resection that provides oncological results comparable to an amputation while still maintaining a functional limb. The patients benefit from the possibility of actively controlling the new knee joint, which allows them to live a very active lifestyle and even enables the opportunity of doing sports like running, soccer or dancing. Furthermore, outcomes of pediatric patients have better functional results compared to endoprosthetic replacement [1, 22–24]. In addition to the excellent functional outcome, Bernthal et al. state that the emotional outcome for the patients has also been very positive even after longer time periods up to ten years and more [25]. The patients usually describe a high quality of life. Borggreve–Van Nes-Winkelmann rotationplasty shows low complication rates and there is no phantom pain since the nerves are spared during this procedure.

A disadvantage that could be considered is the unusual cosmetic appearance of the rotated limb. However, when inserted into a prosthesis, the appearance is similar to any other amputation. All of our seven patients met the first rotationplasty patient in Myanmar, a 7-year-old boy. They saw him walking and playing with his brother and talked to him and his family members. Good preoperative planning and possible exchange with other affected patients and their families are of major advantage in the shared decision-making process leading up to this surgical procedure. A contraindication for Borggreve–Van Nes-Winkelmann rotationplasty is tumor infiltration of the sciatic or tibial nerves which require oncological resection in that case [26]. Nerve resection could lead to motor dysfunction and insensate stump complications [27]. Nevertheless, a successful microvascular rotationplasty with nerve repair in a patient with lower limb sarcoma and vascular as well as neural involvement has been performed and was published in 2016 [28].

In lower-middle income economies, not all surgical treatment options for bone malignancies are available and practical. Safe and low-cost reconstructive options must be established to avoid amputation and ensure limb-salvage treatment whenever possible [20, 29]. This case series of seven patients with osteosarcoma of the distal femur and proximal tibia proves that such a complex surgery as the Borggreve–Van Nes-Winkelmann rotationplasty rotationplasty can be successfully performed even under suboptimal clinical circumstances and limited infrastructure and resources. Due to the reduction of the operating time, blood loss and duration of the stay at an intensive care unit, costs can be minimized. Because of a tent- or V-shaped skin envelope dissection above the femoral amputation site, the preservation of abundant soft tissue for skin adaption and wound closure was enabled. Additionally, the protection of the neurovascular structures of the affected limb optimize the functionality of the reconstruction [30]. The reason why the vascular resection-anastomosis was preferred over the vascular loop-technique is mainly that the vein and artery are located closer to each other wich reduces the postoperative compression of the vessels. For an experienced microsurgeon, this end-to-end anastomosis can be performed safely and fast.

The local orthopedic team of Mandalay Orthopaedic Hospital in Myanmar participated in all seven cases. By this approach, even complex surgical tasks can be performed together, and local surgeons were taught during these procedures in order to ensure a good postoperative follow-up care and treatment. Criticism of case series from lower-middle income economies often arises from the poor follow-up. Our patients were provided with excellent postoperative treatment and follow-up examinations by the participating local orthopedists and oncologists as well as frequent video phone calls from Germany by the corresponding author. Moreover, operated patients are revisited twice a year by German team members.

## Conclusions

A simplified and safe rotationplasty technique was adapted for the insufficient clinical circumstances in lower-middle income economies and was performed successfully with good functional and aesthetic outcomes of seven patients suffering from osteosarcoma.

## Data Availability

All data analyzed in this study are included in the published article or the supplementary files.
